# A Great Light from Philadelphia

**Published:** 1900-08

**Authors:** 


### A GREAT LIGHT FROM PHILADELPHIA.

OUR attention has been directed to an editorial in the Philadelphia *Medical Journal*, July 21, 1900, commenting upon medical matters in this state. The subject is important and further comment seems necessary.

Our impression is that the editor is on his vacation, that the understudy is temporarily indisposed and that the typewriter is new at the work; certainly a more ignorant, illogical and misleading statement upon well-known matters of medical history we have yet to see.

What are we to think of a writer's Americanism who solemnly decides that a state medical society is all wrong which has existed for nearly a century as a delegate or representative body? What a misfortune that the colonies did not have the services of the writer of this so-called comment; and think of what a constitution he could have framed for a government in which every man might expect to be a member of the legislature and every woman a president, or at least a governor!

Again, what are we to think of the sanity of a writer who seriously avers that it is impossible for the members of a county medical society to select certain of its members to represent the society in a central body, or to contend that a central body so constituted could not in any sense be representative of its several constituents ?

How empty of think is one's think-tank who concludes a ponderous paragraph for the enlightenment of the American Medical profession as to the history of the profession of the State of New York and its state society, with the idiotic conclusion that because "The state society now numbers about 600 members out of an estimated total of 9,500 medical practitioners in the state," therefore the former cannot represent the latter.

We feel like offering the writer of this malodorous effusion as a piece of news, the fact that the United States Government exists and its congress of less than 600 members represents its people of some 75 millions.

Finally, what shall we say of the taste of one who writes to illumine the world out of such Egyptian darkness ?

